# Assessment of behavioural problems in preschool and school going children with epilepsy

**DOI:** 10.3934/Neuroscience.2022015

**Published:** 2022-06-01

**Authors:** Harshitha Shanmuganathan, Radha Kumar, D.V. Lal, Chaudhary Devanand Gulab, E. Gayathri, Kesavaraj Pallavi Raja

**Affiliations:** 1 M.B.B.S, M.D. Paediatrics, Fellow in Developmental Paediatrics, Department of Pediatrics, Saveetha medical college and hospital, Thandalam, Kancheepuram, Tamilnadu, 602105, India.; 2 M.B.B.S, M.D. Paediatrics, Professor, Department of Pediatrics, Saveetha medical college and hospital, Thandalam, Kancheepuram, Tamilnadu, 602105, India.; 3 M.B.B.S, D.C.H. Paediatrics, Senior Resident, Department of Pediatrics, Saveetha medical college and hospital, Thandalam, Kancheepuram, Tamilnadu, 602105, India.; 4 M.B.B.S, M.D. Paediatrics, Associate Professor, Department of Pediatrics, Saveetha medical college and hospital, Thandalam, Kancheepuram, Tamilnadu, 602105, India.; 5 M.B.B.S, Post graduate student, Department of Pediatrics, Saveetha medical college and hospital, Thandalam, Kancheepuram, Tamilnadu, 602105, India.; 6 M.B.B.S, Post graduate student, Department of Pediatrics, Saveetha medical college and hospital, Thandalam, Kancheepuram, Tamilnadu, 602105, India.

**Keywords:** drugs, epilepsy, electroencephalography, neuroimaging, seizures

## Abstract

**Introduction:**

Children with epilepsy are at greater risk of developing psychiatric and behavioural disorders such as attention deficit/hyperactivity disorder (ADHD), conduct disorder, autism spectrum disorder (ASD), as well as affective and aggressive disorders than normal children which may affect the well- being and quality of life of the child.

**Aim and Objectives:**

This study aims at identifying behavioural problems in children with epilepsy enabling early diagnosis and intervention. The objectives were to assess the presence and type of behavioural problems in children with epilepsy.

**Methods:**

A prospective cross-sectional study was conducted on children who were diagnosed as epilepsy in two age groups of 1.5–5 years and 6–18 years recruited by non-probability convenience sampling. Data regarding seizure semiology, clinical features and treatment were obtained. Children underwent IQ assessment, electroencephalogram and brain neuroimaging. Child Behaviour Check List (CBCL) was administered to parents or primary caregivers after obtaining informed consent. Results were analyzed for presence of behavioural problems using SPSS-23.

**Results:**

In the study, out of 50 study subjects, 72% were between 6–18 years. 60% children had generalised seizures, 58% children had epilepsy for <2 years and abnormal EEG was present in 80% children. 6% children had behavioural problems and 4% had borderline presentations. Co-relation of behavioural problems with age was statistically significant with p value 0.027. Behavioural problems identified were aggressiveness and anxiety.

**Conclusion:**

Childhood epilepsy is associated with behavioural problems along with other co-morbidities warranting a search during follow-up visits.

**Take-home message:**

Early identification and treatment of behavioural problems in children with epilepsy by periodic assessment during follow up visits, careful selection of combination of drugs and appropriate dose can improve the overall outcome in children taking antiepileptic drugs (AEDs) for epilepsy.

## 1. Introduction

Epilepsy is a common neurological problem in children with majority of cases starting during early childhood. Epilepsy is clinically defined as “at least two unprovoked (or reflex) seizures occurring more than 24 hrs apart” [1]. The overall prevalence of epilepsy in India is 3.0–11.9 per 1,000 population and incidence is 0.2–0.6 per 1,000 population per year which is comparable to that reported from developed countries [2],[3].

Childhood epilepsy may be associated with co-morbidities such as behavioural, cognitive and psychiatric problems [4]. Co morbidities can occur due to underlying structural brain lesion or due to the effect of seizures and inter-ictal discharges on the brain function. Epileptic encephalopathy can occur in West syndrome, Dravet and Lennox-Gastaut syndrome where in children can have intellectual disability, attention deficit hyperactivity disorder or autism. Psychiatric problems like depression and anxiety disorder can occur in children with epilepsy in 35–50% cases [5]. The social stigma which a child may face at home or at school may also affect the psychological behaviour of the child. Temporal lobe epilepsy and frontal lobe epilepsy are associated with agitation and aggressive behaviour. Antiepileptic drugs like sodium valproate and vigabatrin are known to cause aggression and agitation in 1–10% children whereas phenobarbitone produces hyperactivity, behavioural problems and cognitive decline [6].

Behavioural problems in childhood epilepsy may go unnoticed and can have significant impact on the psychosocial development, schooling and quality of life. A pre-existing behavioural problem can get worsened after onset of epilepsy [5]. Behavioural problems are classified as internalizing and externalizing problems. Internalizing problems such as depression, somatic complaints and withdrawal are not emoted by the child to external environment. In externalizing problem, the child emotes to the external environment such as aggressiveness and rule breaking.

There have not been many studies in India highlighting behavioural problems in childhood epilepsy. Hence this study was conducted with the primary objective being to identify the presence of behavioural problems in children with epilepsy from 1 1/2 years to 18 years of age using Child Behaviour Check List and to identify the type of behavioural problem. Early identification and treatment of behavioural problems can improve the overall outcome.

## 2. Methods

***Study design and procedure:*** A cross sectional prospective study was done after obtaining approval from ethical committee under the ethical code SMC/IEC/2018/04/081 where patients were recruited by consecutive, enumerative and non-probability convenient sampling of children in 2 groups over a one year period (1st April 2018–30 March 2019). Seizure semiology details obtained were onset, duration, frequency, loss of consciousness, automatisms and treatment whether on monotherapy or polytherapy anti-epileptic drugs. Children underwent IQ assessment (for children up to 5 years WPPSI-IV (Wechsler Preschool & Primary Scale of Intelligence-IV) and for 6–18 with WISC-IV (Wechsler Intelligence Scale for Children-IV) Indian adaptation), electroencephalogram and neuroimaging. Drug levels were analyzed in multidrug treated epileptics and all those who had recurrence in spite of higher dosage of single drugs to maintain normal levels before behavioural analysis to avoid bias due to higher levels or drug interactions.

***Study participants and sampling:*** Study participants were children aged 1 year 6 months to 18 years, who presented in outpatient department for seizure follow up and were receiving antiepileptic drugs (AED's). Children with epilepsy diagnosed by ILAE 2017 were included and were divided into 2 groups. Total 50 children were enrolled in the following 2 groups. With eight male and six female in group1 (male: female ratio were 1.33:1) and twenty male & sixteen female group 2 (male: female ratio were 1.25:1).

Group 1: Age group between 1 1/2year to 5 years.

Group 2: Age group between 6 year to 18 years.

***Inclusion &exclusion criteria:*** Children of age 1 1/2 years to 18 years who presented with epilepsy diagnosed by the ILAE guidelines (International League Against Epilepsy) 2017 were included in study. Children diagnosed with cerebral palsy, intellectual disability (developmental delay > 2 SD for <5 year and Total IQ < 70 for >5 year), febrile seizures and those unwilling to participate in the study were excluded from the study.

***Study instruments and measures:*** CBCL (Child Behaviour Check List) is a questionnaire based standardized tool used for assessing behavioural problems [7]. CBCL for preschool children (1 1/2–5 years) and school aged children (6–18 years) in local language was administered to the primary caregivers/parents after obtaining written consent. The first page of CBCL records demographic information and ratings of positive behaviours, academic functioning and social competence. The last two pages lists common behavioural problems and the responses are recorded on Likert scale from 0–2. It contains 113 questions for 6–18 yrs of age and 54 questions for 1 1/2–5 yrs of age. The behavioural problem scales can be examined in terms of both broad-band (internalizing, externalizing) and as narrow-band dimensions (e.g. social problems, thought problems, aggressive behavior [9]. The eight domains are anxious/depressed, withdrawn, somatic complaints, social problems, thought problems, attention problems, rule breaking behaviour and aggressive behaviour. For total behavioural problems, T score < 60 are normal range, 60–63 represent borderline scores while scores > 63 represent clinical range. Results was analyzed for correlations between number of AEDs and seizure type and seizure frequency and the presence and type of behavioural problems.

***Ethical aspects:*** The study design was approved by Institutional Review Board (IRB) and after obtaining approval from ethical committee under the ethical code SMC/IEC/2018/04/081 where patients were recruited for the study.

***Data analysis:*** The data analysis was done by SPSS software version 23.0. Since the present study had two groups, paired t test for comparisons among two groups and chi-square test for demographic characteristics was done. Confidence level was set at 95% with P < 0.001 as strongly significant and P < 0.05 as moderately significant. To describe about the data descriptive statistics frequency analysis, percentage analysis were used for categorical variables and the mean & S.D. were used for continuous variables.

## 3. Results

The percentage of boys in the study was 56% compare to that of girls 44%. The frequency of study participants in the range of 1.5 to 5 years was 28% and from 6 to 18 years was 72%.

46% children in age group of 6–18 years and 14% children in the age group 1.5 years to 5 years had generalized type of seizure as most common type of seizure (60%). 26% children in age group of 6–18 years and 14% children in the age group 1.5 years to 5 years had focal type (40%) of seizures. There was no statistically significant difference between seizure type with age of the child (P value–0.368) as shown in Table 1. In our study according to 2017 ILAE classification of seizure type, it was seen that focal with B/L tonic clonic (14%) and focal tonic (14%) were the most common type followed by focal clonic (4%), focal with epileptic spasms (4%), focal myoclonic (2%), and focal atonic (2%). In generalized seizure type generalized tonic/clonic (38%) were the most common type followed by generalized tonic (10%), generalized clonic (4%), generalized non motor typical (4%) and generalized epileptic spasms (4%).

**Table 1.** Comparison between seizure type with age.

Seizure type		Age		Total	Chi^2^-value	P-value
		1.5 to 5 yrs.	6 to 18 yrs.			
GTCS	Count	7	23	30	0.81	0.368 #
	%	14%	46%	60.0%		
Focal	Count	7	13	20		
	%	14%	26%	40.0%		
Total	Count	14	36	50		
	%	28%	72%	100.0%		

# No Statistical Significance at P<0.05 level

Sex distribution of affected children did not show any statistical significance (1.5–5 years male to female ratio was 1.33:1 & between 6–18 years it was 1.25:1). 58% of children had seizure less than 2 year duration, 38% had for 2–6 years and only 4% had seizure duration more than 6 years. The duration of epilepsy when compared to patient age was also found to be not significant statistically (P value-0.165) as shown in Table 2.

**Table 2.** Comparison between duration of epilepsy with Age.

Duration of epilepsy		Age		Total	Chi^2^-value	P-value
		1.5 to 5 yrs.	6 to 18 yrs.			
< 2 yrs.	Count	11	18	29	3.602	0.165 #
	%	22%	36%	58.0%		
2 - 6 yrs.	Count	3	16	19		
	%	6%	32%	38.0%		
>6 yrs.	Count	0	2	2		
	%	0.0%	4%	4.0%		
Total	Count	14	36	50		
	%	28%	72%	100.0%		

# No Statistical Significance at P <0.05 level

84% children received monotherapy AED (66% had sodium valproate acid followed by 14%-Phenytoin, 2% levetiracetam and 2% received cabamazepine) while 16% received polytherapy AED (combination of sodium valproate, phenytoin and levetiracetam in 4%, sodium valproate, levetiracetam and carbamazepine in 4%, sodium valproate and phenytoin in 2% phenytoin and levitiracitam in 2%, carbamazepine and levetiracetam in 2%, sodium valproate and carbamazepine in 2% children). Anticonvulsant therapy when compared to age of child was not significant statistically (P value of 0.083) with single anticonvulsant drug was predominantly used than multiple AED's. On comparing the correlation between number of AED's and seizure type it was found to be statistically non significant (P value = 0.7298). When compared the correlation between number of AED's and seizure frequency it was found to be statistically non significant. (P value = 0.7824).

Abnormal EEG was present in 71.40% of children in age between 1.5–5 years and 83.30% in age between 6–18 years. It was seen that 14.30% of children in age between 1.5–5 years and 13.90% in age between 6–18 years had no abnormal EEG.

MRI brain was done in 52% patients and CT brain was done in 36% children. Neuroimaging was abnormal in 12% of cases and none had neurocutaneous markers or microcephaly. All children had normal intelligence quotient.

While comparing behavioural problems with age, in 6–18 year group 61.1% was normal, 30.6% borderline and 8.3% was abnormal while in 1.5 to 5 years group, 28.6% was normal, 71.4% borderline. The data when analysed was found to be statistically significant with a P value of 0.027 as shown in Table 3.

Internalizing problem and age was correlated in the cases. While analyzing internalizing problem, 30.6 % was borderline and 11.1% were detected as abnormal in the 6 to 18 year age group; where as in group of 1.5 to 5 years 42.9% had borderline internalizing problem. This data was found to be statistically not significant with a P value of 0.367 as shown in Table 4.

**Table 3.** Comparison between Total behavioural problems with Age.

Total behaviour score CBCL			Age		Total	Chi^2^-value	P-value
			1.5 to 5 yrs	6 to 18 yrs			
Total	Normal	Count	4	22	26	7.229	0.027 *
		%	28.6%	61.1%	52.0%		
	Borderline	Count	10	11	21		
		%	71.4%	30.6%	42.0%		
	Abnormal	Count	0	3	3		
		%	0.0%	8.3%	6.0%		
Total	Count	14	36	50		
	%	100.0%	100.0%	100.0%		

* Statistical Significance at P<0.05 level

**Table 4.** Comparison between Internalizing problem with Age.

Internalizing problem		Age		Total	Chi^2^-value	P-value
		1.5 to 5 yrs	6 to 18 yrs			
Normal	Count	8	21	29	2.007	0.367 #
	%	57.1%	58.3%	58.0%		
Borderline	Count	6	11	17		
	%	42.9%	30.6%	34.0%		
Abnormal	Count	0	4	4		
	%	0.0%	11.1%	8.0%		
Total	Count	14	36	50		
	%	100.0%	100.0%	100.0%		

# No Statistical Significance at P<0.05 level

While correlating the age of the child with externalizing problem 38.9% were borderline and 8.3% were abnormal. While in the group of 1.5 to 5 years, 64.3% had borderline externalizing problem. This data was found statistically not significant with a P value of 0.203 as shown in Table 5.

**Table 5.** Comparison between Externalizing problem with Age.

Externalizing problem		Age		Total	Chi^2^-value	P-value
		1.5 to 5 yrs	6 - 18 yrs			
Normal	Count	5	19	24	3.191	0.203 #
	%	35.7%	52.8%	48.0%		
Borderline	Count	9	14	23		
	%	64.3%	38.9%	46.0%		
Abnormal	Count	0	3	3		
	%	0.0%	8.3%	6.0%		
Total	Count	14	36	50		
	%	100.0%	100.0%	100.0%		

# No Statistical Significance at P<0.05 level

Among the eight domains in CBCL, 10% had features of anxiety, 4% were withdrawn, 2% had somatic complaints, 2% had thought problems and 10% had aggressiveness. None in the study had social problems, attention problems and rule breaking behaviour.

## 4. Discussion

This study was intended to bring out the behavioural changes in children with epilepsy with specific focus on effect of antiepileptic drugs. Epilepsy in children is associated with higher incidence of behavioural problems compared with normal children and has a negative psychosocial impact[8]. A review of recent studies showed that the prevalence of mental health problems in school going children varies from 6.33% to 43.1% in Indian context [9]. In the present study the proportion of boys (56%) was found to be more than that of girls (44%) with (M:F is 1.27:1), and 28% children were within the range of 1.5 to 5 years whereas 72% were between 6 to 18 years with mean age of 11.25 years. In both the age groups, more children received monotherapy compared to polytherapy. In another study there were no significant differences as regard to total behavioural problems between children on monotherapy as compared to polytherapy in both younger as well as older age groups [10]. A relatively higher percentage of children with below average IQ had total behavioural problems in comparison to those who had average IQ in both younger as well as older age group but the differences were found to be insignificant [10],[11].

Among 1.5 to 5 years group receiving anticonvulsant therapy, monotherapy was administered in 78.6% cases and polytherapy in 21.4% cases whereas in 6 to 18 years mono drugs was administered in 86.1% of cases and poly drug in 16% of cases and the comparison was found to be statistically not significant.

A significant effect of age of onset, frequency of seizures and number of antiepileptic drugs in relation to behavioural problems have been reported earlier [12]. A cross-sectional study done by Om P Mishra et al. on children with epilepsy and normal controls found that younger age of onset, and frequency of seizures were significantly associated with behavioural problems. In addition, duration of disease in different age groups and anti-epileptic drugs in older children also affected the internalizing problems. However, no difference in behavioural problems was observed between mono and polytherapy [10]. In contrast, effect of polytherapy over behavioural problems was found by Datta et al. in their patients with epilepsy [13].

There are multiple factors which affect the behavioural domains in children with epilepsy. Further it is likely that the child's psychological perception of the disease situation, especially in older children, could be another contributing factor to the patient's behaviour during the course of illness. Present study mandated maintaining normal blood levels of anticonvulsants before behaviour assessment in both poly and monotherapy groups, probably resulting in lesser number of significant behavioural issues. Thus, use of minimum number of antiepileptic drugs for seizure-control should be aimed, to minimize the occurrence of behavioural impairment in these children.

In our study the parents reported significantly more internalizing as opposed to externalizing behaviour problems, a pattern reported previously. For each scale, internalizing and externalizing problem scales and the total score, scores can be interpreted as falling in the normal, borderline or clinical behaviour, any score that falls below the 93^rd^centile is considered normal, scores between the 93-37^th^centile are borderline clinical, and any score above 97^th^ percentile are in the clinical range. In our study, internalizing problems were present in 8% and 34% had borderline internalizing problems. The internalizing CBCL scales include social withdrawal, somatic complaints, and anxiety/depression. In contrast, no group differences were observed for any of the externalizing behaviour problem scales (aggressive, rule breaking behaviours). In our study externalizing problems in clinical range was 6% and borderline clinical were 46% and children with total behavioural problems in clinical range were 6% and borderline clinical were 42%. In summary, relative specificity was exhibited in regard to behavioural problems as opposed to an endorsement of global behavioural and social impairment. The study by Del Canto et al. showed that 50% of 159 children with epilepsy had emotional and behavioral problems and internalizing problems were present more than externalizing problems as seen in our study. Presence of both internalizing and externalizing problems lead to increased parental stress, which was significantly related to emotional and behavioral symptoms in the children [5]. A study conducted by Çelen Yoldaş T et al. on 43 preschool children with new onset epilepsy who were on followup showed that compared to controls , there was increase in internalizing, externalizing, and total problems in the children with epilepsy [14].

Symon M. Kariuki et al. conducted a study in Kenya to record behavioral problems in children with epilepsy compared with controls using child behavioral questionnaire. A significantly greater proportion of children with epilepsy (49% vs 26% of controls) were reported to have behavioral problems [15]. The study done by Karanja et al. on 177 children with epilepsy aged 6–12 years showed that total emotional and behavioral symptoms were present in 46% of cases, which was mainly aggressive behavior, attention issues ,social problems, withdrawal and depression [16].

In a cross-sectional study to screen for behavioural problems by Dora Novriska et al., comprising of 47 children with epilepsy and 46 children without epilepsy aged 3–16 years, behavioral problems were seen in 19.1% of children with epilepsy and 2.2% of children without epilepsy (PR 8.8; 95% CI 1.16 to 66.77; P = 0.015) [17].

Behavioural problems and cognitive impairment are commonly seen in children with epilepsy. In sub-Saharan Africa, little is known about these comorbidities particularly their relationships with socioeconomic features [18].

Behavioural status shows a different pattern of association with clinical seizure correlates. Other than age at seizure onset, frequency of seizures during the past year emerged as the strongest and most consistent predictor of behavioural difficulties.

There was significant association of increased frequency of seizures in the past year with seven of eight behavioural scales (the lone exception was social problems). In addition, higher frequency of seizures was also significantly related to the total behaviour problems scale and the total internalizing and social competence scales. These findings are consistent with other reports [18].

It remains to clarify the underlying mechanism(s) for the association between seizure frequency in the past year and behaviour problems. Seizure frequency could impact behaviour in the context of the nature and pattern of cerebral disturbance underlying the seizures, parental distress about seizure activity, or through increased exposure to psychosocial factors such as the stigma, social isolation, and family dysfunction that may accompany increased seizure frequency. Most studies to date, including the present one, have relied primarily upon parent report of behavioural adjustment. This method of ascertainment introduces the possibility of parental reporting bias and other mitigating factors which are associated with seizure activity but which do not directly reflect neurophysiological features of seizure activity. Nevertheless, it is of interest to note that parental ratings were not elevated across all dimensions of behavioural adjustment. Future studies that collect behaviour rating from other sources (e.g. teachers) would be useful in distinguishing between the possible mechanisms for the relationship between seizure activity and behavioural adjustment. It should be pointed out that there was a significant overall relationship between the integrity of neuropsychological status and adequacy of behavioural functioning. Specifically, more impairment in overall neuropsychological status was modestly but significantly associated with increased total behavioural problems. This relationship appears to be reliable as it has been reported previously but appears to be of modest explanatory power [19],[20].

The findings point to areas of cognitive vulnerability and difficulties in behavioural adjustment that are observed in children with seizures even when compared with an age and sex-matched sibling control group. These findings raise important issues and considerations for early clinical assessment and identification of these potential problems so that appropriate programs of intervention can be initiated [21]. Internalizing problems like anxiety disorder in the child may lead to increased parental concern leading to dysfunctional parent-child interaction [22].

Future research work needs to expand on our understanding of the underlying neurophysiological and social mechanisms that play a part in producing these behavioural problems.

## 5. Conclusion

The present study shows that behavioural co-morbidities differ in children with epilepsy from different age-groups, with externalizing behaviour in younger children and both internalizing and externalizing behaviour in older age-group and can be assessed reliably with the use of CBCL during follow-up. Due attention should be given by pediatricians for recognition of behavioural problems in children with epilepsy by periodic assessment during follow up visits, careful consideration of dose, selection and combination of drug and if abnormalities are detected, may need counseling, supportive psychotherapy and also adjustment on behalf of parents.
